# Saunders’ Medical Hand Atlases

**Published:** 1901-08

**Authors:** 


SAUNDERS' MEDICAL HAND ATLASES.—ATLAS AND EPITOME OF OPHTHALMOSCOPY AND OPHTHALMOSCOPIC DIAGNOSIS. By Professor Dr. O. Haab, Director of the Eye Clinic in Zurich. From the third revised and enlarged German edition. Edited by Geo. E. DeSchweinitz, Professor of Ophthalmology, Jefferson Medical College, Philadelphia. With 152 colored lithographic illustrations and 85 pages of text. Philadelphia and London: W. B. Saunders & Co. 1901. Price, $3.00 net.

Students and physicians who are interested in the study of the eye-ground will find Professor Haab's atlas of inestimable value. All of the ophthalmoscopic images and other illustrations contained in this work were prepared by the author himself, and they constitute one of the best and most complete collections of the kind in existence.

